# LS CE-Chirp^®^ vs. Click in the neuroaudiological diagnosis by ABR^☆^

**DOI:** 10.1016/j.bjorl.2016.04.018

**Published:** 2016-05-31

**Authors:** Michelle Cargnelutti, Pedro Luis Cóser, Eliara Pinto Vieira Biaggio

**Affiliations:** a Universidade Federal de Santa Maria (UFSM), Distúrbios da Comunicação Humana, Santa Maria, RS, Brazil; b Clinica Coser de Otorrino, Santa Maria, RS, Brazil; c Universidade Federal de Santa Maria (UFSM), Santa Maria, RS, Brazil

**Keywords:** Hearing, Electrophysiology, Auditory brainstem response, Audição, Eletrofisiologia, Potenciais evocados auditivos

## Abstract

**Introduction:**

The chirp stimulus was developed seeking to counterbalance the delay of the sound wave on its journey through the cochlea, allowing the hair cells to depolarize at the same time. The result is a simultaneous stimulation providing better neural synchrony and, consequently, the recording of responses with greater amplitudes.

**Objective:**

To compare the absolute latency of waves I, III and V, the interpeak intervals I–III, III–V and I–V, amplitude values of wave V and its association with the amplitude of wave I, and the interaural difference V–V in the auditory brainstem response (ABR) using Click and LS CE-Chirp^®^ stimuli to determine whether the responses evoked by LS CE-Chirp^®^ could be applied to neuroaudiological diagnosis.

**Methods:**

Cross-sectional study with 30 normal-hearing individuals. The parameters used were: intensity of 85 dBnHL, alternating polarity; 17.1 stimuli/s and 100–3000 Hz filters.

**Results:**

The absolute latencies of waves I, III and V observed with LS CE-Chirp^®^ and click did not show significant differences. Significantly higher amplitudes of wave V were observed with the LS CE-Chirp^®^. The interaural difference between the wave V latencies between stimuli showed no significant difference.

**Conclusion:**

The LS CE-Chirp^®^ stimulus was shown to be as efficient as the click to capture ABR at high levels of stimulation, with the advantage of producing greater-amplitude V waves.

## Resumo

**Introdução:**

O *chirp* foi desenvolvido buscando compensar o atraso da onda sonora em seu trajeto pela cóclea, possibilitando que as células ciliadas despolarizem ao mesmo tempo. O resultado é uma estimulação simultânea proporcionando uma melhor sincronia neural e consequentemente o registro de respostas ocorre com maiores amplitudes.

**Objetivo:**

Comparar a latência absoluta das ondas I, III e V, os intervalos interpicos I–III, III–V e I–V, os valores de amplitude da onda V e sua relação com a amplitude da onda I, e a diferença interaural V–V no Potencial Evocado Auditivo de Tronco Encefálico, utilizando os estímulos Clique e LS CE-Chirp^®^, a fim de determinar se as respostas evocadas por LS CE-Chirp^®^ poderiam ter aplicabilidade no diagnóstico neuroaudiólogico.

**Método:**

Estudo transversal com 30 indivíduos normo-ouvintes. Os parâmetros utilizados foram: intensidade de 85 dBnNA, polaridade alternada; 17,1 estímulo/s e filtros de 100–3000 Hz.

**Resultado:**

As latências absolutas das ondas I, III e V observadas com LS CE-Chirp^®^ e clique não tiveram diferenças significativas. Amplitudes significativamente maiores da onda V foram observadas com o LS CE-Chirp^®^. A diferença interaural entre as latências da onda V entre os estímulos, não mostrou diferença significativa.

**Conclusão:**

O estímulo LS CE-Chirp^®^ mostrou-se ser tão eficiente quanto o clique na captação do Potencial Evocado Auditivo de Tronco Encefálico, em níveis elevados de estimulação, com a vantagem de produzir ondas V de maior amplitude.

## Introduction

The auditory brainstem response (ABR) examination records responses of the neural pathway, and is useful in the assessment of the auditory system integrity. It is clinically used to estimate hearing thresholds of adults and infants^1^ and to detect nervous system disorders at the peripheral and central level.^2^ It is historically recorded with a transient click stimulus with a rapid onset and short duration (100 μs). The composition of this stimulus is broadband, with maximum peak power in the regions from 1000 to 4000 Hz.^3^ Considering the cochlear tonotopy when the click stimulus is presented at the cochlea, each region of the basilar membrane is stimulated, one after another, from the base to the apex. Thus, the transient stimulus stimulates the high-frequency region earlier than those corresponding to low frequencies. Because the low-frequency components provide delayed response peaks to be added to the response to high-frequency components, much information is lost in the overall response sum. Therefore, the results of the excitation of different nerve fibers at different times decreases the neural synchrony, which is necessary to evoke a hearing potential.^4^

From this perspective, in order to compensate for the sound wave delay on its trajectory through the cochlea, researchers have designed a stimulus which they named *chirp*. This stimulus has a duration up to 10.33 ms, which is much longer than the click. It was designed so that low frequencies are presented before the high ones, so that different regions of frequencies reach their specific place at the basilar membrane simultaneously. Consequently, in this way, the cochlear neural response is more synchronized and a greater amplitude of wave V is displayed at the ABR recording.^4^, ^5^, ^6^

Several chirp models have been proposed and tested,^4^, ^6^, ^7^, ^8^, ^9^ and one of the researchers’ aims is to find the most adequate stimulus for wave V recoding of the ABR at low intensities.

When studying^7^ several chirp models, chirp researchers found that short-duration chirps were more effective at high intensities and long-duration chirps were more effective at low intensities, and that the stimulus intensity is directly related to the wave V amplitude. Given these facts, Claus Elberling and his group of researchers concluded that the CE-Chirp^®^ model, based only on the duration of time required for the sound wave to travel through the different cochlear frequency regions, is inadequate to generate a robust ABR response in adults with normal hearing. Therefore, they improved the stimulus design and created a model called “direct approach”.^10^ This new model took into consideration the sound travel time in the cochlea and also different levels of intensity. Unlike the CE-Chirp^®^ and others that preceded it, this new *chirp* has no fixed duration at all intensities; it was created with variable duration for each stimulus intensity, thus comprising twenty stimuli of different durations that change every 5 dB. This new stimulus was called level specific CE-Chirp^®^ (LS CE-Chirp^®^) and has been tested, purporting to overcome the limitations of the CE-Chirp^®^,^11^ which evoked only a very small wave V amplitude and did not allow the identification of waves I and III at high stimulation intensities.

The few literature articles on this stimulus^11^, ^12^ only discussed its advantage in detecting wave V with high amplitude even at high intensity and mentioned that only the I and III waves were also found in all 10 assessed subjects. Therefore, we decided to measure the values of absolute latencies of waves I, III and V, the interpeak intervals I–III, III–V and I–V, the amplitude values of wave V and its association with the amplitude of wave I, and the V–V interaural difference in the auditory brainstem response (ABR) using Click and LS CE-Chirp^®^ stimuli to determine whether this new stimulus could be applied to neuroaudiological diagnosis by ABR.

## Methods

This was a cross-sectional study with a descriptive design, associated to a wider project approved by the ethics committee on human research, according to opinion N. 610506, on April 8, 2014, and CAAE 14804714.2.0000.5346. It should be noted that it followed Resolution N. 66/12, which deals with human research. All subjects were informed of the study aim, as well as the procedures involved and all subjects signed the free and informed consent form, agreeing to participate in this study.

Inclusion criteria were: individuals with no history of otological or neurological disease and audiometric thresholds ≤25 dBHL for the 250–8000 Hz frequencies. The study included 30 individuals (18 women and 12 men) aged 12–42 years with normal hearing.

The ABR records with Click and LS CE-Chirp^®^ stimuli were performed using the Eclipse EP25 ABR system^®^ equipment, manufactured by Interacoustics A/S, Denmark. The parameters used for the recordings were: alternating polarity, presentation rate of 17.1 stimuli/s; passband filter of 100–3000 Hz for the Click and LS CE-Chirp^®^ stimuli, 10-millisecond (ms) window, stimulation using ER-3A insert earphones at the intensity of 85 dBnHL, with no additional filtering being made after response acquisition.

The subjects were placed on a stretcher, so that they were comfortably lying down. The electrode attachment sites were cleaned with Nuprep abrasive paste and the electrodes were attached to the skin. The reference electrodes were placed on the right (A2) and left (A1) mastoids and the active (Fz) and ground (Fpz) electrodes on the forehead. The recording only started with electrode impedance below 3 kΩ.

The ABR recording was monaural, starting at the right ear with the Click stimulus and, after that, the recording was performed in the left ear. Subsequently, the LS CE-Chirp^®^ stimulus was used, also starting with the right ear and finally, in the left ear.

The recording was interrupted with a minimum of 1000 stimuli and the duplication of each record was carried out to ensure the reproducibility and reliability of the waves.

The presence/absence of waves I, III and V was analyzed, comparing the absolute latency values of waves I, III and V; amplitude values of wave V and wave I and the values of interpeak latencies (IPLs) I–III, III–V, I–V and the interaural difference V–V between the LS CE-Chirp^®^ and click stimuli.

To compare the latency and amplitude variables between the stimuli, Student's *t* test was used for independent samples. At the analysis of the variables interaural difference V–V and V/I ratio, the Mann–Whitney *U* test was used to compare the click and LS CE-Chirp^®^ stimuli.

For all analyses, the significance level was set at 0.05 (5%) and confidence intervals were built with 95% statistical confidence.

## Results

When comparing the results of the absolute latencies of waves I, III and V and the amplitude of wave V between the right and left ears, it was observed that the differences between the ears were not significant for both Click and LS CE-Chirp^®^ stimuli. Thus, the values of both ears were always considered for data analysis, doubling the sample size.

Table 1 compares the absolute latencies of waves I, III and V between the stimuli, at an intensity of 85 dBHL, with no statistically significant difference in the comparison between the Click and LS CE-Chirp^®^ stimuli.

**Table 1:** Descriptive statistics for the absolute latencies of waves I, III and V of ABR with Click and LS CE-Chirp^®^ stimuli, at 85 dBnHL in normal hearing adults (*n* = 60).

Variables	Stimuli		*p*-Value
	Click	LS CE-Chirp^®^	
Wave I	1.29 (±0.09)	1.29 (±0.13)	0.921
Wave III	3.42 (±0.15)	3.42 (±0.18)	0.978
Wave V	5.27 (±0.18)	5.19 (±0.24)	0.885

ABR, auditory brainstem response; dBnHL, decibel for normal hearing level.

The mean values of Interpeak latencies I–III, III–V and I–V for the stimuli used in this study are shown in Table 2.

**Table 2:** Descriptive statistics for I–III, III–V and I–V interpeak latencies of ABR between Click and LS CE-Chirp^®^ stimuli at 85 dBnHL in normal hearing adults (*n* = 60).

Variables	Stimuli	
	Click	LS CE-Chirp^®^
	Mean (SD)	Mean (SD)
I–III	2.13 (±0.14)	2.13 (±0.14)
III–V	1.85 (±0.18)	1.77 (±0.22)
I–V	3.98 (±0.21)	3.90 (±0.25)

ABR, auditory brainstem response; dBnHL, decibel for normal hearing level; SD, standard deviation.

Table 3 shows that at the intensity assessed, 85 dBnHL, the amplitude of wave V in ABR recordings with the LS CE-Chirp^®^ stimulus was greater than the amplitude of the recordings with the click stimulus, with a significant difference.

**Table 3:** Descriptive statistics for wave V amplitude at ABR with Click and LS CE-Chirp^®^ stimuli, at 85 dBnHL in normal hearing adults.

	Stimuli		*p*-Value
	Click	LS CE-Chirp^®^	
Wave V amplitude	0.50 (±0.19)	0.61 (±0.23)	0.004

ABR, auditory brainstem response; dBnHL, decibel for normal hearing level.

When comparing the amplitude values of V/I ratio between the stimuli, statistically significant higher values were elicited by the LS CE-Chirp^®^ stimulus (Table 4).

**Table 4:** Descriptive statistics for the V/I ratio of ABR with Click and LS CE-Chirp^®^ stimuli, at 85 dBnHL in normal hearing adults.

	Stimuli		*p*-Value
	Click	LS CE-Chirp^®^	
V/I ratio	1.63 (±1.3)	2.07 (±1.3)	0.004

ABR, auditory brainstem response; dBnHL, decibel for normal hearing level.

Table 5 shows that no statistically significant difference was observed when comparing the interaural latencies of wave V between the stimuli used in this study.

**Table 5:** Descriptive statistics between V–V between Click and LS CE-Chirp^®^ stimuli at ABR, at 85 dBnHL in normal hearing adults.

	Stimuli		*p*-Value
	Click	LS CE-Chirp^®^	
	Mean SD	Mean SD	
V–V	0.08 (±0.09)	0.10 (±0.09)	0.273

ABR, auditory brainstem response; dBnHL, decibel for normal hearing level; Sd, standard deviation.

According to the results obtained in this study, it is suggested that the values shown in Table 6 can be used as maximum values of normality (mean value plus 2 standard deviations) for neuroaudiological diagnosis, for both the click and the LS CE-Chirp^®^ stimuli, presented at 85 dBnHL.

**Table 6:** Maximum values for absolute wave and interpeak latencies of ABR for neuroaudiological diagnosis with both stimuli – Click and LS CE-Chirp^®^, at 85 dBnHL.

Latencies	Wave I	Wave III	Wave V
	1.55 ms	3.79 ms	5.67 ms

Intervals	I–V	I–III	III–V	V–V
	4.40 ms	2.40 ms	2.20 ms	0.28 ms

ABR, auditory brainstem response; dBnHL, decibel for normal hearing level; ms, milliseconds.

**Figure 1:**
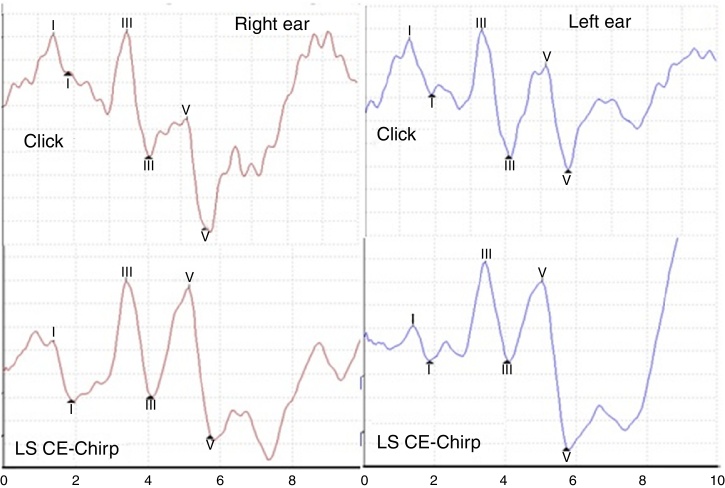
Example of ABR recording with Click and LS CE-Chirp^®^ stimuli in one subject. Observe the much higher amplitude of wave V in response to the LS CE-Chirp^®^ and similar latencies in response to Click and LS CE-Chirp^®^ on both sides.

Fig. 1 shows the ABR recordings registered with the LS CE-Chirp^®^ and click stimuli in one of the study subjects.

## Discussion

This study showed that in all ABR recordings the waves I, III and V were identified both with Click and LS CE-Chirp^®^ stimuli, at a stimulation level of 85 dBHL. Another study^12^ recorded ABR in normal-hearing adults, using the stimuli: Click, CE-Chirp^®^ and LS CE-Chirp^®^, and observed that at 80 dBnHL, all peaks of waves I, III and V were identified when using the Click and LS CE-Chirp^®^ stimuli. When using the CE-Chirp^®^ stimulus at this stimulation level, wave I was not observed in any of the records, wave III appeared in 35% and wave V in 90% of recordings. Another investigation^13^ compared ABR records using click and CE-Chirp^®^ in normal-hearing adults. The researchers analyzed the presence/absence of waves I, III and V at 80 dBnHL and concluded that waves I and III tended to disappear when using the CE-Chirp^®^ stimulus. The LS CE-Chirp^®^ stimulus was not used in the study by Lewis & Roberts. When comparing values for absolute latencies between the stimuli, shorter values were observed for the CE-Chirp^®^ stimulus, when compared to the click stimulus.

However, in a study recording ABR at 80 dBnHL, wave V latencies were longer for the LS CE-Chirp^®^ stimulus when compared to Click. According to the study researchers, this result was due to the fact that in the LS CE-Chirp^®^, most frequency components, reach the cochlea 1.5 ms later than the corresponding components in the Click and, thus, the latency at ABR with the LS CE-Chirp^®^ stimulus was higher when compared to Click.^12^

However, the commercial version of LS CE-Chirp^®^, when using the Eclipse EP25 ABR system^®^ equipment manufactured by Interacoustics, changed the way LS CE-Chirp^®^, is presented, using as point zero of the stimulus the location of the chirp corresponding to the 2500 Hz frequency, instead of the location of the final frequency of 10,000 Hz of the LS CE-Chirp^®^ used in research. This change resulted in responses with latencies equal to those obtained with the click in its several intensities. Regarding the amplitude of wave V, we found significantly higher values in the recordings with the LS CE-Chirp^®^ stimulus. These results are consistent with a study that compared the amplitude between LS CE-Chirp^®^ and Click and concluded that the LS CE-Chirp^®^ stimulus in ABR recordings provides significantly higher amplitudes when compared to Click, at higher levels of stimulation (80 dBnHL).^12^

The current study corroborates several studies^4^, ^13^ that also observed a higher response to wave V amplitude when using the chirp stimulus in ABR recordings in normal-hearing adults.

It is worth mentioning that studies using the LS CE-Chirp^®^ stimulus are carried out in individuals with cochlear and retrocochlear pathology to better assess their contributions to clinical practice.

## Conclusion

The LS-CE Chirp^®^ stimulus is as efficient as the click in obtaining waves I, III and V of the auditory brainstem response test, at high levels of stimulation. This stimulus may be useful in neuroaudiological diagnosis because unlike CE-Chirp^®^ that most of the time evokes only waves III and V, the LS-CE Chirp^®^ stimulus evokes the three waves needed for this type of diagnosis, with the added advantage that wave V has greater amplitude than when evoked by the click.

## Conflicts of interest

The authors declare no conflicts of interest.
